# Professionalism in dentistry: deconstructing common terminology

**DOI:** 10.1038/s41405-022-00105-9

**Published:** 2022-07-23

**Authors:** Andrew Trathen, Sasha Scambler, Jennifer E. Gallagher

**Affiliations:** Faculty of Dentistry, Oral & Craniofacial Sciences, Centre for Host Microbiome Interactions, King’s College London, London, UK

**Keywords:** Dentistry, Occupational health

## Abstract

**Background:**

There is a social expectation that dentists demonstrate professionalism. Although the General Dental Council puts it at the heart of their regulatory agenda, there is not yet consensus on the meaning and implications of the term.

**Objective:**

To explore practising dentists’ understanding of the character traits commonly associated with professionalism and what these mean in practice.

**Method:**

Constructivist grounded theory was employed throughout this study. Qualitative, in-depth interviews were conducted with dental professionals in England recruited through theoretical sampling to saturation point. Interviews used a topic guide informed by the literature, and analysis was conducted through constant comparison during data collection.

**Results:**

The study found that traits commonly associated with professionalism in the literature were difficult for dentists to define clearly or operationalise in a clinical setting. There was disagreement over how some traits should be understood, and it was unclear to participants how, or indeed if, the listed traits were directly relevant to practice in their current form.

**Conclusion:**

Rather than expecting unconditional adherence to an externally imposed definition, further exploration is required to understand how health professionals make sense of professionalism by reference to their lived experiences and worldviews.

**In Brief:**

- Institutional expectations of professionalism, defined through character traits and behaviours, do not appear to map neatly on to the experiences of dental professionals.
- Straightforward, apparently uncontroversial terms elicited a wide range of responses, including disagreement. This brought in to question whether achieving consensus is possible.
- Analysing how our respondents understood the terms by reference to the meanings they constructed from lived experience offers deeper insights.

## Introduction

The healthcare perspective on professionalism recognises an ethical duty to the people that the professions serve and building a shared set of values is considered an important part of fulfilling this duty [1]. One of the persistent and intractable difficulties of trying to use professionalism in this way is that it is dependent on reaching consensus over what the values and behaviours that constitute professionalism should be. Reaching a shared understanding has been difficult, but there is broad agreement that some basic principles such as putting the needs of the patient first, learning, and being virtuous should be taught, with many also citing the need for assessment and measurement [2].

As in other areas of healthcare, professionalism in dentistry has received significant attention through the years [3–7], with efforts usually directed at trying to clarify what the term means, and how it should be operationalised by practitioners, educators, and regulators [8, 9]. The work reflects a similar long-term effort in medicine to deepen our understanding of what the term means, and how it is valuable to patients and healthcare professionals.

Most substantive efforts at understanding and promoting professionalism in healthcare have taken the approach advocated by Inui [1]. This involves formulating lists of attributes and codifying them into a set of guidelines for professionalism [10–12], and the discourse around this tends to be institutionalised [13]. Educators at universities and working groups at national associations and colleges expend resources on the professionalism project. Over time, this has led to the development of a range of definitions based on character traits seen as related to professionalism in a health environment [14].

These lists of character attributes portray the ‘ideal’ healthcare professional. This functionalist [15], aspirational, *ideal-archetype* model of professionalism has cross-cultural applicability [16–18], and is useful for teaching undergraduates, as they will have very limited experience of practice and will be developing their professional worldviews both formally and informally [19, 20].

However, as a recently graduated dentist gains experience in practice outside the relatively protected world of their training institution, they may see professional ideals learned as an undergraduate through a different lens. A host of factors will influence how they understand their professional role and the duties they have, beyond the formalised standards and guidelines set by universities, Royal Colleges, and the General Dental Council.

If we try to remedy this by moving away from the ideal-archetype model, things become more nebulous. It potentially leaves professionalism as “*an umbrella that covers a host of complex, somewhat interconnected important ideas that comprise a tangle of elements”* [21]. Bundled into this are less virtuous issues linked to preserving the income and status of the profession. This corresponds to decades of sociological literature that understands professionalism in terms of power relations and as a method of labour division [22–24]. High income, autonomy and status require that the profession commits to giving something back to society in return [25], and one view is that institutionalised professional codes are an effort to demonstrate that they can be trusted to do so. The Royal College of Physicians 2005 definition of professionalism [11], incorporates character traits which include:

- Integrity
- Compassion
- Excellence
- Reliability
- Altruism
- Continuous improvement
- Working in partnership with members of the wider healthcare team.

This list, they explicitly state: *“underpins the trust the public has”*.

In a 2009 paper [8], Trathen and Gallagher argue that this definition, although originally developed within medicine, could potentially be transferable to dentistry because it comes from a UK institution, and represents the views of leaders and policy influencers within the field of healthcare. As such it provided a convenient starting point to develop further research into the subject. The paper also proposed that the business angle of healthcare, neglected by research on professionalism in medicine in the UK context, needed to be examined in light of the way dental care is organised in the UK, with co-payments and a mix of private and NHS practice. This is of particular relevance if, as Welie suggests, dentistry as a profession and as a business are not commensurate [26].

The Royal College of Physicians (RCP) list provided a way to initiate a discussion and allowed us to explore the extent to which the discourse of organisations, and the character trait lists that emerge from them, correlate to the ways dentists understand and enact professionalism in practice [27]. Taking this as a starting point, this paper presents the findings from a qualitative study exploring dentists’ understanding of these character traits. These findings form a single strand of a far more expansive grounded theory study which aimed to answer the research question, *what does dental professionalism mean in practice?*

## Method

Constructivist grounded theory (CGT) [28] was employed to conduct and analyse qualitative, depth interviews with dental professionals in England. A stratified sample of dental professionals practising around London and the north of England were approached to participate in one-to-one interviews by email. All interview participants worked within the UK dental system, a cross section of National Health Service (NHS) primary and secondary care and private practice.

The initial phase of sampling purposively aimed to vary role, practice type (NHS/Private), and patient demographics to represent the different contexts in which dentistry is delivered. Theoretical sampling was employed for later interviews to ensure saturation of categories and develop the emergent theory [29, 30]. Three significant phases of theoretical sampling involved firstly dentists and dental care professionals (DCPs) to explore and contrast perspectives, before refocusing towards private practitioners who had reservations about how NHS care was delivered in the UK. A later phase of theoretical sampling sought dentists who moved away from a primary care environment to explore their reasons for doing so. Data were collected between 2012 and 2015, with a final period of collection in 2017.

We adopted the CGT method as described by Charmaz [28, 31, 32]. This method is well suited for generating novel theory, and ensures it emerges from and remains linked to the data [33]. The constructivist component emphasises the socially constructed frameworks that individuals use to make sense of their lived experiences. Interviews used a topic guide informed by the literature [34]. This was piloted with two respondents and revised in light of the results for subsequent interviews. Initial questions explored respondent understandings of terms used in the RCP definition of professionalism [11]. Interviews were audio recorded with field notes taken, and the data transcribed. Duration ranged between approximately 45 and 80 min. Data were recorded and coded using NVivo 10 and Word software. Transcriptions were subject to a three-stage coding process. Initial coding was done tentatively line by line, converting data into gerunds (‘doing’ words). Next, focused coding highlighted the most important codes and filtered irrelevant ones. The final stage was theoretical coding, at which point codes were grouped together to generate higher level categories from which theory could be derived [28]. Ethics permission was granted by the King’s College London Research Ethics Committee (reference BDM/11/12-27, LRS-17/18-5297).

In total, 24 dental professionals consented to participate in one-to-one interviews. The age of respondents ranged from 27 to 57 years; 14 were male and 10 female; 9 were early, 11 mid and 4 in late career stages. Two informants were DCPs and the remainder dentists. Of these, two worked in secondary care (one senior consultant and one senior house officer (SHO) in restorative dentistry and dental public health) and they received fixed salaries. The remainder were general dental practitioners, 7 exclusively private and 13 NHS (also doing variable amounts of private work). Two had the experience of working in corporate-owned practices. One was in the process of changing career from dentistry to medicine, moving from MBBS to foundation training. 18 respondents were based in greater London, two of whom operated from central London postcodes. Two came from surrounding counties of Hertfordshire and Surrey. Three came from further north in England: Nottingham, Sheffield, and Newcastle.

One had training and experience in an EU country before becoming a dentist in a high-needs area of London. Larger practices indicate they had five or more surgeries within the practice.

An overview of respondents’ details is presented in Table 1.

**Table 1:** Respondent characteristics.

Respondent designation	Gender	Career stage	Position
D1	F	Mid	DCP
D2	M	Late	DCP
S1	M	Late	Secondary care
S2	M	Early	Secondary care
G1	F	Early	NHS
G2	M	Early	NHS
G3	M	Late	NHS
G4	M	Mid	NHS
G5	M	Mid	NHS
G6	M	Mid	Private
G7	M	Mid	Private
G8	F	Mid	Private
G9	F	Early	NHS
G10	M	Early	Private
G11	F	Mid	Private
G12	F	Late	NHS
G13	F	Mid	NHS
G14	M	Mid	Private
G15	M	Mid	NHS
G16	F	Early	NHS
G17	F	Mid	Private
G18	M	Early	NHS
G19	F	Early	NHS/medic
G20	M	Early	NHS

*D* dental care professional; *S* Secondary care; *G* general dental practitioner.

## Results

The results presented here reflect the responses of working dental professionals to the RCP character traits defined as comprising professionalism within a healthcare setting. The traits were integrity, compassion, excellence, reliability, altruism, continuous improvement, and working with the health team. Each trait was presented within the interview and participants were asked what the term meant to them and how and if it was relevant to their practice. Each term is taken in turn below and the views of participants are illustrated with quotes. Quotes are attributed as: respondent designation/male or female/workplace setting/transcription line number (e.g., G1f/NHS/l.100).

### Integrity

There was some uncertainty around the word integrity and its meaning. Most were familiar with the term, but some were either unsure about its exact meaning or how to interpret it in the light of professionalism. All of the participants who were comfortable with the term saw it as a character trait. For some this was related predominantly to trust and honesty:

*You need professional integrity because you need your patients to completely trust in you. I guess your whole character is a build-up of this. From your appearance to the way you talk, your accent, everything is really, will support integrity (G18m/NHS/l.179−185)*

Alongside trust, the term honesty repeatedly occurred when participants tried to explain how they understood integrity, and in many cases, the terms appeared to be almost synonymous. Descriptions such as ‘transparent’, ‘reputation’ and ‘being a person of their word’ were used to describe integrity in this way.

For other participants integrity was related to being reliable; doing what you say you will do, sticking to your values. In the words of one participant:

*being true to yourself… and your values (G8f/private/l.775)*.

She also pointed out that to have integrity the values you stand by should be good, and that may not necessarily be so.

*…you could have really bad values and be true to them wouldn’t you and you could be a scoundrel so yes no. (G8f/private/l.778)*

Two respondents provided examples to illustrate their perception of integrity as ‘doing what is best for your patient’. In the context of private practice, they used examples where choices could be made in clinical situations that would all be reasonable. They felt that where one option would be financially more rewarding, choosing the other when they felt it best was a good example of integrity. There appeared to be competing demands on the respondents’ decision making.

*Let’s say you need a crown … shall we go for white crown or gold crown or something like that. Now I might get more money if I get a white crown off you but it’s my obligation to you to say gold is the best material… give you all the information to not do that (G10m/private/l.548−556)*

Whilst no informants considered integrity to be a bad thing per se, there were varying views about its relevance to professionalism or to dentistry.

*It’s not the utmost thing I would say is the key thing for a dentist to be*. *(G3m/NHS/l.116−118)*

*I would say it’s important I would not say it’s the most important, but I do think it’s important…I’ve heard it used, but not really that much in dentistry I guess. (G16f/NHS/l.109−116)*

What is clear from this is that there is some confusion about what integrity means and how it might apply to dental practice. The implication of this is that if ‘integrity’ is a social or regulatory demand, its interpretation in practice is unlikely to be consistent or straightforward.

### Compassion

There was a broad, shared understanding of the term compassion. Views on its applicability to dentistry were varied, however, ranging from a suspicion that it was too strong a word to apply to dental practice, to a belief that a dentist would be in the wrong job if they did not have it.

Pain was frequently mentioned when discussing the need for compassion. This involved the importance of recognising the pain that somebody else is in, and seeking to alleviate it, and tied compassion to empathy.

*If a patient comes in in pain or if a patient comes in that is upset with the work that you’ve done or something like that, you need to be compassionate in putting their feelings and their situation first before yourself. (G10m/private/l.963−968)*

This was also perceived as potentially problematic by some participants because it added complexity to the issue of discussing fees; there were several ethical factors pulling in different directions when navigating such situations.

*The difficult point actually on that one is when a patient comes in with raging toothache and all they want is for you to take away their pain and there is something that’s very uncomfortable about talking money before taking away their pain. Because it sounds like well if you give me this much money, I’ll take away your pain. Whereas actually the reality is you’re going to take away their pain and sometimes in that situation you almost have a kind of feel your way about whether they can accept your* *fees and you usually end up under-charging them if you don’t think they can because you have to get rid of the pain first. (G11f/private/l.425−447)*

This is a clear example of the divergence between medicine and dentistry in the UK context, with the co-payment element of dentistry requiring conversations about money which do not routinely take place in medicine. The outcome of this was that, whilst compassion itself was seen as a virtue, when considered in the context of professionalism many participants felt that it could have a detrimental effect if taken too far, for both dentist and patient.

*You need a degree of compassion, but I think some people can get a bit over the top… it’s a fine balance I think.” (G2m/NHS/l.321−325)*

Another respondent felt that compassion was too strong a word for dentistry, and that empathy was better. She felt that this implied being able to consider other people’s feelings without becoming too drawn into their circumstances.

### Altruism

Altruism proved to be one of the least well-understood terms amongst participants in this study. In interviews where they were unfamiliar with the word, altruism was explained as ‘putting the needs of others before your own’. Generally, the examples given were things such as staying later to help emergency patients or making an extra call to a colleague to double check a patient is being managed well.

Some people felt that altruism meant doing something for no reward, which was not considered appropriate. Participants expected to be ‘paid properly’ for what they do.

*I obviously believe that professionalism should be paid properly, and the word ‘altruism’ is more doing it without any rewards. I do believe that it should be rewarded, but yeah, I agree with the word altruism, yeah. (G3m/NHS/l.66−71)*

This led to accounts, as above, where answers were contradictory, suggesting a discordance between actual beliefs and what participants felt they ‘should’ say. When it was put to people that one possible form of altruism would be to lower fees for patients, the consensus was that this was not something that should be expected of a dentist. It was repeatedly explained that dentists have to make money and run successful businesses.

*And it’s a business at the end of the day […], we all run a business and that, I suppose is an individual thing. (G10m/private/l.994−1004)*

Building on this, the idea that working in the NHS is ‘charity’, and thus altruistic, emerged repeatedly in the accounts of participants.

*I mean, to be honest I would think that the NHS is a bit of altruism […] Because I would say that the NHS doesn’t financially benefit dentists as much as private healthcare does. (G20m/NHS/l.349−354)*

This idea is repeated almost exactly by another respondent.

*Well, the NHS system […] sometimes you are having to do more work than probably you are paid for I guess so sometimes that’s when I’d probably use that word altruistic because that’s when you’re putting the needs of others before your own, so that is where I’d probably apply that word. (G16f/NHS/l.219−228)*

Again, this raises interesting points of divergence between dentistry and medicine around the roles of business and remuneration in the delivery of care.

### Continuous improvement

Of all the characteristics discussed, the only one to have consistent agreement was the concept of continuous improvement. No clarifications were asked for and there was agreement that it is important. Variation came in how the respondents felt it should be put into practice.

Change was an important theme that emerged when discussing continuous improvement. Some examples were given of older dentists who had not kept up with contemporary issues, which was seen as a problem.

*I think at the very least pick up dental update [a dental magazine] come on. It costs 80 pound a year. (G2m/NHS/l.583−589)*

One participant described how she took over from another dentist who tended not to do things she considered fairly basic. She described the surprise of the patients at the changes she made.

*They’re like, Mr X never did that. They love him. They’re not upset with me, they’re almost intrigued. They’re like, “oh what’s she going to do next?” (G9f/NHS/l.1095−1100)*

She suggested that patients were quite forgiving of their old dentist, suggesting that their relationship with him was more important than the technical aspects of the examination they received.

*…he’s actually almost become a permanent member of their extended family and they love him even though deep down, they don’t think he’s probably kept up with [contemporary dental care]… well, they still think he’s professional. (G9f/NHS/l.1095−1100)*

There is an irony that the one term that was uncontroversial to dentists (despite different understandings and degree of perceived importance) was perhaps less important to patients than some of the other traits that had little consensus.

### Excellence

The idea or trait of excellence created some confusion. Much of this stemmed from the perceived logical inconsistency of using this term as a professional attribute, as well as questions over what precisely excellence was supposed to cover and what it might look like. One participant responded to the use of the term with a barrage of questions seeking to clarify what exactly was being referring to.

*You can’t measure it. Having excellent habits would lead … does it lead to excellence? What is excellence? It’s outstanding, isn’t it … how can you all be outstanding? Kind of a contradiction … We all have to have a minimum level of excellence. Is it excellent to pass exams? (G9f/NHS/l.1233−1241)*

If excellence is taken as a superlative it was noted that it would then, by definition, be impossible for everyone to reach an excellent standard; excellence implies being better than average. Thus, a more pragmatic attitude was thought to be acceptance that an average dentist will be average, but that they should aspire to excellence and work towards it, making efforts to continually improve and avoid stagnating.

*If the dentist doing the work strives to make it excellent then I think that’s sort of really the major part (G3m/NHS/l.364−367)*

Many respondents felt that excellence was an ‘ideal’ or something that was ‘aspirational’ rather than a required endpoint for all, thus making the inclusion of excellence in the definition of professionalism problematic without some kind of clarification.

### Partnership with the healthcare team

‘Partnership with the healthcare team,’ needed further clarification for nearly every interview. Even when clarification was provided, however, there was disagreement about what a ‘good’ partnership would look like. Participants tended not to assess the appropriateness of the words themselves, but rather discussed experiences of working with others. For some, an emphasis on leadership and hierarchy emerged; for others, they described the importance of collaboration and non-hierarchal relationships. For some, interpersonal relationships were most important.

### Reliability

Finally, all respondents felt that reliability was an important part of professionalism, but some suggested it was less important than other traits.

*…to a certain extent you of course need a bit of reliability, you can’t just not be there when you say you will be there and things like that for patients. On the other hand, I wouldn’t say that if you’re not reliable* *then you’re not professional. I don’t think that comes in (G3m/NHS/l.647−654)*

Again, reliability was understood in different ways with some participants relating it to personal behaviours such as punctuality and following through on your word, while others linked it solely to the consistency of one’s clinical work.

What can clearly be seen from the responses to this list is that all but one trait is understood in a variety of, sometimes contradictory, ways. In addition, the relative importance of the traits varies between participants.

This has profound implications for discussions about professional practice in a dental setting. It seems that an externally imposed set of traits and behaviours is conceptually challenging to apply to real-world practice, because a shared framework of understanding and application does not appear to exist.

## Discussion

As a grounded theory study, it is important to be clear on what type of knowledge this research is and is not capable of providing us, and what sort of ‘reality’ we are trying to capture. Constructivist grounded theory (CGT) was consciously selected with these epistemological and ontological questions in mind.

Ontologically, our presupposition is that when asking what professionalism means in practice, the answer will not be something that exists independently from the mind; we can only interpret the question by reference to the viewpoints of individuals. Epistemologically, the design of the study does not seek to achieve a fully representative sample of these individuals through geography, sample size etc; rather it followed the needs of the emergent theory. Instead of attempting to acquire generalisable truths, it sought to create an explanatory framework that more comprehensively elucidates certain elements of the social world, specifically, what dental professionalism means in practice.

The findings of this study demonstrate the limitations of using solely word-based character traits to understand and define professionalism within dentistry. Even words which superficially seem uncontroversial elicited a wide range of, sometimes opposing, views.

A typical criticism levelled at the professionalism discourse is the contention that the words used to mark out the character traits are so vague and abstract that they are almost impossible to disagree with [21]. But the findings suggest that this isn’t the case. There *is* disagreement, leading to a much more intractable problem - even where there was a degree of shared understanding of the traits, there was disagreement over whether the words should be part of a definition of professionalism at all. For example, participants did not just disagree on the meaning of the term ‘altruism’, they also disagreed on whether such a concept is appropriate to the practice of dentistry.

Part of this undoubtedly arises from a fundamental problem of creating shared intersubjective understanding of language. The CGT method used for this study provides particularly useful insight into the nature of this problem. CGT aims to get close to how respondents understand their social world. By presenting them with institutionally selected terms that are deemed to form professionalism, declared by fiat, we are forcing the participants to make sense of words and ideas constructed by someone else. When we discussed these words, participants had to *deconstruct* them, in a literary sense.

In literary criticism and philosophy, deconstruction unpicks language that has been formulated into a closed configuration or is axiomatic, particularly when it concerns ideas that are purported to be absolute, fixed, and necessary [35, 36]. The institutional RCP definition of professionalism fits these criteria.

Although we are not suggesting that the respondents are deliberately deconstructing these words with a literary precision, the process of discussing the words in the semi-structured interview format continually subverted the idea that these words are mutually intelligible, let alone agreed upon. Respondents were only able to create meaning by incorporating them into an experiential framework comprised of their working experiences, beliefs, and worldviews—they constructed their *own* meanings. They each had their own *social-professional constructs*. And there was much more variation than we anticipated.

This process is recognisable through the paradigm of *social constructivism*, the idea that knowledge and understanding for an individual develops from their interactions with culture and society. It is a foundational principle of the ‘constructivist’ grounded theory method used for the study.

The limitations of the word-based, functionalist understanding of professionalism [37] emerged early in the research process, and the insight began to explain why efforts to define professionalism over years and decades have struggled. Words used to describe professionalism are not, and likely could not, be agreed upon to make a conventional operational definition.

Although the social-professional constructs of the respondents needed to be internally coherent (i.e., a person will need to avoid the dissonance of conflicting beliefs/ideas), the constructs of any two individuals are going to be different. The lived experiences that are drawn upon to create these constructs are always going to be unique.

Tackling our research question by trying to understand the respondents’ social-professional constructs allowed us to develop a much more far-reaching, context-based conception of what professionalism means. This is the core of an explanatory grounded theory that is capable of accounting for the lack of agreement observed around institutionally defined terms.

One accepted limitation of this study is the use of a single definition provided by the Royal College of Physicians in 2005 [11]. However, there was a clear rationale for doing do. First, it is, as an ideal-archetype model, based on listed traits characteristic of the structural-functionalist definitions common to professionalism discourse in healthcare and the earlier sociology of the professions [37]. Second, it came from a UK institution and had national relevance. Third, it represented the contemporary views of leaders and policy influencers within the field of healthcare at the time when we embarked on the research. Thus, in summary, we consider that it has provided a useful starting point to develop further research on this important subject for the dental context [8].

Although it is merely the initial research findings, rather than the full grounded theory in this paper, the findings we present endorse concerns articulated by other authors who suggest the need for alternative approaches to the ‘immutable’ language used in the discourse of professionalism [38–40]. Our study raises the possibility that reaching a shared understanding may be insurmountably difficult if that understanding is contextually and cognitively bounded.

## Conclusion

A different approach to understanding and operationalising professionalism is needed. One line of reasoning in the literature suggests that professionalism is not ‘*an absolute but constructed in the interaction of individual and context’* [41]. Whilst researchers have moved discussion forwards and recognise the need for context [40, 42], we are still largely reliant on functionalist lists of character traits in discussions of professionalism that describe an ideal-archetype. These remain at the core of curricula designed to teach future dentists what is expected of them as professionals.

However, understanding practitioners working outside of this protected academic environment brings in to question how useful this idealistic approach actually is, as it does not map neatly on to the varied and often surprising data we collected, and it does not appear to reflect the lived realities of our respondents.

Complexity and context are key to developing a theory that is useful, intellectual and practical for professionalism in healthcare and dentistry [43]. Engaging with the social-professional constructs which individuals rely on to understand their social world is one route to achieving this.

## References

[1] Inui TS. A Flag in the Wind: Educating for Professionalism in Medicine. Washington, DC: Association of American Medical Colleges, 2003.

[2] Zijlstra-Shaw S, Roberts TE, Robinson PG (2013). Perceptions of professionalism in dentistry - a qualitative study. Br Dent J..

[3] Dunning JM (1982). Dentistry at the crossroads: a study of professionalism. Am J Public Health.

[4] American Board of Internal Medicine. Project professionalism. Philadelphia, PA: American Board of Internal Medicine, 1995.

[5] Swick HM (2000). Toward a normative definition of medical professionalism. Acad Med..

[6] D’Cunha J (2010). Professionalism in medicine: are we closer to unifying principles?. Semin Thorac Cardiovasc Surg..

[7] Phillips R, Bazemore AW, Newton WP, Byyny R. Medical professionalism: a contract with society. Pharos. 2019:2–7.

[8] Trathen A, Gallagher JE (2009). Dental professionalism: definitions and debate. Br Dent J..

[9] Zijlstra-Shaw S, Robinson PG, Roberts T (2011). Assessing professionalism within dental education; the need for a definition. Eur. J. Dent. Educ..

[10] American Board of Internal Medicine Foundation, American College of Physicians-American Society of Internal Medicine, European Federation of Internal Medicine. (2002). Medical professionalism in the new millennium: a physician charter. Ann Intern Med..

[11] Royal College of Physicians. Doctors in society: Medical professionalism in a changing world. Report of a Working Party of the Royal College of Physicians of London. London: RCP, 2005. 16408403

[12] American Dental Education Association. ADEA Statement on professionalism in dental education. ADEA; 2009.

[13] Hafferty F, Levinson D (2008). Moving beyond nostalgia and motives: towards a complexity science view of medical professionalism. Perspect Biol Med..

[14] Birden H, Glass N, Wilson I, Harrison M, Usherwood T, Nass D (2014). Defining professionalism in medical education: a systematic review. Med Teach..

[15] Saks M. Professions and power: A review of theories of professions and power. *In* Dent M, Bourgeault IL, Denis J-L, Kuhlmann E, (eds). *The Routledge Companion to the Professions and Professionalism*. New York, NY: Routledge, 2016. 85–91.

[16] Chandratilake M, McAleer S, Gibson J (2012). Cultural similarities and differences in medical professionalism: a multi-region study. Med Educ..

[17] Haque M, Zulkifli Z, Haque SZ, Kamal ZM, Salam A, Bhagat V (2016). Professionalism perspectives among medical students of a novel medical graduate school in Malaysia. Adv Med Educ Pract..

[18] Jacob L, Ramachandran G (2019). Professionalism in medical school. Quest Int J Med Health Sci..

[19] Masella RS (2006). The hidden curriculum: value added in dental education. J Dent Educ..

[20] van Mook WN, de Grave WS, van Luijk SJ, O'Sullivan H, Wass V, Schuwirth LW (2009). Training and learning professionalism in the medical school curriculum: current considerations. Eur J Intern Med..

[21] Erde EL (2008). Professionalism’s facets: ambiguity, ambivalence, and nostalgia. J Med Philos..

[22] Larson MS. *The rise of professionalism: Monopolies of competence and sheltered markets*. New Brunswick, NJ: Transaction, 2013.

[23] Abbott A. *The system of professions: An essay on the division of expert labor*. Chicago, IL: University of Chicago Press, 1988.

[24] Macdonald KM. *The sociology of the professions*. London: SAGE, 1995.

[25] Holden ACL (2017). Dentistry’s social contract and the loss of professionalism. Aust Dent J..

[26] Welie JV (2004). Is dentistry a profession? Part 3. Future challenges. J Can Dent Assoc (Tor.).

[27] Shirley JL, Padgett SM. An analysis of the discourse of professionalism. *In* Wear D, Aultman J M, (eds). *Professionalism in medicine: critical perspectives*. Boston, MA: Springer, 2006. 25–41.

[28] Charmaz K. *Constructing grounded theory*. 2nd ed. London: SAGE, 2014.

[29] Draucker CB, Martsolf DS, Ross R, Rusk TB (2007). Theoretical sampling and category development in grounded theory. Qual Health Res..

[30] Conlon C, Timonen V, Elliott-O’Dare C, O’Keeffe S, Foley G (2020). Confused about theoretical sampling? Engaging theoretical sampling in diverse grounded theory studies. Qual. Health Res..

[31] Charmaz K. The power and potential of grounded theory. University of Leicester: Medical Sociology Online; 2012.

[32] Gibson B, Hartman J. *Rediscovering grounded theory*. London: SAGE, 2014.

[33] Foley G, Timonen V (2015). Using grounded theory method to capture and analyze health care experiences. Health Serv Res..

[34] King N, Horrocks C. *Interviews in qualitative research*. London: SAGE, 2010.

[35] Turner C (2013). Deconstructing transitional justice. Law Crit..

[36] Turner C. *Jacques Derrida: Deconstruction*. Critical Legal Thinking. 2016. Available at: https://criticallegalthinking.com/2016/05/27/jacques-derrida-deconstruction (accessed 16 July 2020).

[37] Saks M. Defining a profession: The role of knowledge and expertise. *Professions and Professionalism*. 2012;2.

[38] Wear D, Nixon LL (2002). Literary inquiry and professional development in medicine: against abstractions. Perspect Biol Med..

[39] Wear D, Kuczewski MG (2004). The professionalism movement: can we pause?. Am J Bioeth..

[40] Wynia MK, Papadakis MA, Sullivan WM, Hafferty FW (2014). More than a list of values and desired behaviors: a foundational understanding of medical professionalism. Acad Med..

[41] Castellani B, Hafferty FW. The complexities of medical professionalism. *In* Wear D, Aultman J M, (eds). *Professionalism in medicine: critical perspectives*: Springer, 2006. pp 3-23.

[42] Zijlstra-Shaw S, Roberts T, G Robinson P (2016). Evaluation of an assessment system for professionalism amongst dental students. Eur J Dent Educ..

[43] Morrow G, Burford B, Rothwell C, Carter M, McLachlan J, Illing J. Professionalism in healthcare professionals. London: Health & Care Professions Council, 2014.

